# Characterization of Free and Porous Silicon-Encapsulated Superparamagnetic Iron Oxide Nanoparticles as Platforms for the Development of Theranostic Vaccines

**DOI:** 10.3390/medsci2010051

**Published:** 2014-02-20

**Authors:** Charles M. Lundquist, Christopher Loo, Ismail M. Meraz, Jorge De La Cerda, Xuewu Liu, Rita E. Serda

**Affiliations:** 1 Nanomedicine and Biomedical Engineering, The University of Texas School of Medicine, Houston, TX 77030, USA; 2 Department of Nanomedicine, Houston Methodist Research Institute, 6670 Bertner Ave, Houston, TX 77030, USA; 3 Small Animal Imaging Facility, University of Texas MD Anderson Cancer Center, 1515 Holcombe Blvd., Houston, TX 77030, USA

**Keywords:** porous silicon, iron oxide, magnetic resonance, antigen, adjuvant, vaccine

## Abstract

Tracking vaccine components from the site of injection to their destination in lymphatic tissue, and simultaneously monitoring immune effects, sheds light on the influence of vaccine components on particle and immune cell trafficking and therapeutic efficacy. In this study, we create a hybrid particle vaccine platform comprised of porous silicon (pSi) and superparamagnetic iron oxide nanoparticles (SPIONs). The impact of nanoparticle size and mode of presentation on magnetic resonance contrast enhancement are examined. SPION-enhanced relaxivity increased as the core diameter of the nanoparticle increased, while encapsulation of SPIONs within a pSi matrix had only minor effects on T2 and no significant effect on T2* relaxation. Following intravenous injection of single and hybrid particles, there was an increase in negative contrast in the spleen, with changes in contrast being slightly greater for free compared to silicon encapsulated SPIONs. Incubation of bone marrow-derived dendritic cells (BMDC) with pSi microparticles loaded with SPIONs, SIINFEKL peptide, and lipopolysaccharide stimulated immune cell interactions and interferon gamma production in OT-1 TCR transgenic CD8^+^ T cells. Overall, the hybrid particle platform enabled presentation of a complex payload that was traceable, stimulated functional T cell and BMDC interactions, and resolved in cellular activation of T cells in response to a specific antigen.

## 1. Introduction

Cellular uptake of microbes by dendritic cells (DC) is accompanied by engagement of pattern recognition receptors [1, 2]. Activation of these receptors induces phagocytosis and expression of genes that cause maturation of the cell and activation of anti-microbial events, thereby inducing innate immunity. Particles carrying pathogen-associated molecular patterns, e.g., lipopolysaccharide (LPS), similarly induce phagocytosis and activate antigen presenting cells (APC). We previously demonstrated that DC incubated with porous silicon (pSi) particles presenting LPS and antigen (*i.e.*, ovalbumin peptide SIINFEKL) are actively engaged by T cells obtained from C57BL/6-Tg(TcraTcrb) (a.k.a. OT-1) mice expressing a transgenic TCR for recognition of major histocompatibility complex (MHC) class I-presented antigen. The stimulated DC have enhanced migration to the draining lymph node and elevated expression of MHC and costimulatory molecules, as well as increased secretion of proinflammatory cytokines. While these early studies relied on *ex vivo* modification of APC with fluorescent tracers, particle-based vaccines that incorporate contrast agents can be used experimentally or clinically for noninvasive detection by magnetic resonance imaging (MRI) to track migration of particles, or their cell-based carriers, to lymphatic tissue for modulation of immune responses.

Contrast in MR images is due to differences in signal relaxation rates that are unique to the physical and chemical characteristics of biological tissues [3]. Return to the equilibrium occupancy of spins following excitation is referred to as longitudinal relaxation, and is characterized by an exponential time constant generally referred to as T1. Attenuation of the excited signal due to the dephasing of spins is known as transverse relaxation, which is referred to as T2. Inhomogeneities in the magnetic field that are not compensated for through the use of a spin-echo pulse sequences lead to shorter transverse relaxation time constants, denoted as T2* to differentiate intrinsic tissue relaxation from that induced by magnetic field inhomogeneities. Exogenous contrast agents act to reduce these time constants; for example, an agent that reduces T1 increases signal intensity by accelerating the return to equilibrium between excitations, while an agent that reduces T2 or T2* causes the signal to attenuate more quickly after excitation, leading to negative (dark) contrast [4, 5]. The relaxation time (T2, T2*) and concentration of contrast agent are related by the following equations [6]:

$$R_{1,2}=R_{1,2}^{0}+r_{1,2}\cdot C$$

$$R_{1,2}^{0}=\frac{1}{T_{1,2}^{0}}R_{1,2}=\frac{1}{T_{1,2}}$$

where *T*_1,2_ is the relaxation time at a given concentration of contrast agent, $T_{1,2}^{0}$ is the relaxation time of the medium with contrast agent removed, *C* is the concentration of contrast agent, and *r*_1,2_ is the concentration-independent relaxivity of the contrast agent under study. The term *T*_1,2_ in the above equation is referred to as the relaxation rate.

Superparamagnetic iron oxide nanoparticles (SPIONs) shorten T2 and T2* due to their large magnetic moments and the large magnetic field gradients that surround them [7]. The ability of SPIONs to enhance contrast depends on their size, surface properties, aggregation and compartmentalization [8–10]. For homogeneously dispersed small particles, the motional averaging regime (MAR theory) predicts that transverse relaxivities, r_2_ and r_2_*, increase with increasing core size [11]. That is, the ability of SPIONs to act as contrast agents to lower the T2 relaxation time of tissues is enhanced by increasing the particle core size due to increased magnetic susceptibility. It has been shown that increasing the core size results in a higher induced magnetization at saturation and thus a higher magnetic susceptibility, calculated as *M =χ • H*where M is the induced magnetization, H is the field strength of the magnet, and χ is the suseptibility of the magnetic particle [11, 12]. Above a certain size, further increases in core size fail to enhance relaxation rates based on the existence of strong dipolar fields surrounding the particle, limiting the impact of water diffusion and leading to a plateau in relaxation rate. For smaller particles, r_2_ and r_2_* should be nearly equal based on dependence on diffusion effects, however, for larger particles, the static dephasing regime (SDR theory) predicts that R_2_* increases with increasing local magnetic dose, meaning that particle aggregation leads to increases in R_2_* [8]. In this study, we compare transverse relaxivities of SPIONs with increasing particle core size ranging from 5–30 nm; and we examine the impact of SPION confinement within a pSi matrix on magnetic properties [13]. This data was presented in an abstract in the Proceedings of the ASME 2nd Global Congress on NanoEngineering for Medicine and Biology [13].

Size and aspect ratio of particles are important properties that impact particle biodistribution, cellular interactions, and cellular internalization [14–16]. With respect to subcutaneous inoculation, particles traffic to lymphatic tissue (*i.e.*, lymph nodes) in a size-dependent manner, with large particles being engulfed by peripheral APCs at the site of injection, and small nanoparticles being internalized by resident APCs following cell-free trafficking [17]. In this study, we explore accumulation of free and pSi-encapsulated SPIONs in the spleen following intravenous injection to study the impact of nanoparticle and microparticle trafficking to lymphatic tissue via the vascular system. We further compare T cell immunity stimulated by pSi microparticles presented as single verses hybrid particle complexes.

## 2. Experimental Section

### Materials

SPIONs were purchased from Ocean Nanotech, LLC (Springdale, AR, USA). The iron oxide nanocrystals had an amphiphilic polymer coating containing terminal amine groups attached to polyethylene glycol linkers, or carboxylic acid moieties. C57BL/6 and C57BL/6-Tg(TcraTcrb) (OT-1) mice were obtained from Charles River Laboratories, Inc. (Wilmington, MA, USA) and The Jackson Laboratory (Bar Harbor, ME, USA), respectively.

### Fabrication of pSi Microparticles

1 µm (diameter) × 400 nm (thickness) discoidal pSi microparticles were fabricated in the Microelectronics Research Center at The University of Texas at Austin by combination of electrochemical etching and standard photolithography. Using heavily doped p++ type (100) silicon wafers with resistivity of 0.005 ohm-cm (Silicon Quest, Inc., Santa Clara, CA, USA) as the silicon source and 1 HF (49%): 3 ethanol solution as etchant, the anodic etching process started with applying a 2.3 mA/cm^2^ current for 20 s to form a low porosity mechanical layer, then the current was ramped to 14 mA/cm^2^ in a time course of 15 s, and kept at 14mA/cm^2^ for 45 s to form the porous layer with 60 nm average pores. A current of 76 mA/cm^2^ was applied for 6 s to form the high porosity release layer. Following the formation of porous film, a 40 nm SiO_2_ layer was deposited by Low Pressure Chemical Vapor Deposition at 400 °C. Standard photolithography was then used to pattern an array of 1 µm circles over the SiO_2_ capped porous film using a contact aligner (K.Suss MA6 mask aligner) and NR9-500P photoresist (Futurrex Franklin, NJ, USA). The pattern was transferred into the porous film by Reactive Ion Etch in CF_4_ plasma (Plasmatherm BatchTop, 15 sccm CF4, 100 mTorr, 200 W RF). The capping SiO_2_ layer was removed in 49% HF, and the microparticles were released from the substrate in isopropanol by sonication.

### Surface Modification of pSi Microparticles

pSi microparticles were oxidized with piranha solution (1 volume H_2_O_2_ and 2 volumes of H_2_SO_4_) with heating to 110–120 °C for 2 h as previously described [18]. Oxidized pSi microparticles were washed in IPA, and then suspended in IPA containing 0.5% (v/v) APTES (Sigma-Aldrich, St. Louis, MO, USA) for 0.5–2 h at 35 °C and 1300 rpm. The APTES-modified pSi microparticles were washed in IPA and dried in a desiccator. Volumetric particle size, size distribution and count were obtained using a Multisizer 4 Coulter^®^ Particle Counter (Beckman Coulter, Fullerton, CA, USA). The morphology of the particles was verified by scanning electron microscopy (SEM).

### MRI Phantoms

MR phantoms were prepared in thin wall NMR tubes with 10 mm outer diameters (Wilmad Labglass, Vineland, NJ, USA). Samples of free SPIONs with core sizes of 5, 10, 20, and 30 nm were prepared by adding a 0.005, 0.01, 0.03, and 0.05 mg Fe/mL to a 1% agarose suspension to create unique sample tubes for each core size. Iron content was verified using a Prussian blue assay as described later. Sample sets of 4–5 phantoms, including a 1% agarose only control, were moved into a specially-machined tube holder which was placed into a 300 mL cylindrical polypropylene tube. The tube was then filled with relaxed water (doped with 0.1% Magnevist v/v) to reduce proximity to and interference from air interfaces, capped, sealed, and imaged. MR imaging of phantoms was carried out in an actively-shielded 7 Tesla Biospec USR70/30 (Bruker Biospin MRI, Billerica, MA, USA) small animal MRI system equipped with a 30-cm bore, imaging gradients with a 6-cm free bore, and a linear ^1^H birdcage-style volume resonator with 35 mm inner diameter. A three-plane, T_2_-weighted fast spin-echo sequence (TE = 50 milliseconds, TR = 2,000 milliseconds, ETL = 8,) was used to confirm sample placement. Transverse relaxation time constants (T_2_*) were measured using a multi-echo gradient-echo sequence (minimum TE = 1.5 ms, echo spacing 3.25 ms, 24 echoes, TR = 4,000 ms, 30° excitation).

### Loading SPIONs into pSi Microparticles

Experiments comparing relaxivity values for free verses pSi-encapsulated 30 nm SPIONs were performed by loading 1 × 10^8^ pSi microparticles with serial dilutions of 5, 2.5, 1.75 mg Fe/mL. Loading was accomplished by means of capillary action by adding SPION suspensions to dry pSi microparticles, briefly sonicating, and incubating for 15 min at room temperature. The microparticles were then washed with 50 µL of DI water and centrifuged at 5,000 G × 10 min. The supernatant with excess unloaded SPIONs was pipetted off and the pSi microparticles were desiccated under vacuum. The loading and wash steps were then repeated and the resulting loaded pSi microparticles were suspended in 1% agarose for imaging. The iron content in the samples was verified using inductively coupled plasma-atomic emission spectroscopy (ICP-AES) as described below.

### ICP-AES Analysis of Iron in Phantoms and Tissue Samples

To verify the iron content of the resected tissues, spleen were dehydrated with ethanol and dried overnight. Samples were weighed and placed into nickel crucibles, which were heated in a 500 °C oven for several hours. Once fully ashed, tissue remains were collected by washing the crucibles with 1% spectrosol™ solution and sonicating, up to a final volume of 5 mL. Samples were centrifuged at 4,200 RPM for 10 min to remove ash aggregates from the solution and 4 mL of the dissolved sample was moved to a new conical tube. 1mL of a 1% spectrosol solution, plus 50 µL of 100 mg/L yttrium standard solution, was added to each tube and were analyzed using iron standard concentrations of 25, 75, 100, 250, and 500 ppb, one blank sample of 1% spectrosol solution, and one quality control internal standard containing 250 ppb of iron. Samples were analyzed with a Varian Vista AX at a power of 1 kW, plasma flow set to 15 L/min, auxiliary flow of 1.5 L/min, and a nebulizer flow of 0.75 L/min, with 5 replicate readings at 15 seconds between each reading. The concentration of iron in agarose phantoms was measured by serial dilution of the phantom contents with a reference range of 25–500 ppb. A final volume of 5 mL of 1% spectrosol plus sample solution was used.

### Prussian Blue Assay for Iron Content

To verify total iron content of phantoms used for particle size comparisons, 5 µL samples of 5, 10, 20, and 30 nm stock solutions, as well as Ferric iron (III) standards at 1.5, 1, 0.5, 0.1, and 0.05 mg/mL were incubated with 120 µL 6N HCl for 2 h at 60 °C in triplicate. Iron was then oxidized using 0.1 mg/ml ammonium persulfate (BioRad, Richmond, CA, USA) and the color reaction was initiated by adding 125 µL of a 5% K_4_[Fe(CN)_6_]·3H_2_O (Sigma-Aldrich) for 10 min. Following incubation, sample absorbance was determined at 690 nm using a SPECTRA max M2 plate reader (Molecular Devices, Sunnyvale, CA, USA). A standard curve was then generated using iron III hexahydrate (Sigma-Aldrich) and the iron concentration of SPION preparations was calculated. Cells treated with particles for 24 h (20 pSi particles/cell or 2 µg/mL Fe) were labeled by incubation of cells in acid solution containing potassium ferrocyanide for 30 min at 37 °C.

### *In Vivo* Imaging Experiments

All animal procedures were performed in accordance with recommendations by the National Institutes of Health in the guide “Care and use of Laboratory Animals.” All protocols were reviewed and approved by the Institutional Animal Care and Use Committee at The Methodist Hospital Research Institute (TMHRI), Houston, Texas (Protocol #AUP-1010-0028 and AUP-0311-0012; OLAW Assurance #A4555-01) and at the University of Texas MD Anderson Cancer Center. Time-dependent accumulation of SPIONs in the spleen of female nu/nu mice (Charles River, Wilmington, MA, USA) was determined by intravenous injection of 100 µg SPIONs (Fe content; 20 nm core) in 100 µL PBS. Spleens were excised from mice at 2 and 24 h, suspended in agarose phantoms, and T_2_-weighted images were acquired using a 7 Tesla scanner as described previously.

To determine the impact of pSi encapsulation of SPIONs on contrast enhancement in live animals, mice were intravenously injected with PBS or 100 µg of 30 nm SPIONs (free or pSi-encapsulated). T_2_-weighted images were taken 24 h post-injection. Signal intensities were determined for 5 regions of interest (mid-section of 5 slices), with normalization against a reference phantom containing 0.01 mg/mL SPIONs or against the psoas muscle. Spleens were isolated and embedded in paraffin 24 h after pSi-SPION injections. Tissue sections were labeled with Prussian blue and Nuclear Fast Red as previously described [19].

### Preparation of Adjuvant Particles

An aqueous 1 mg/mL solution of lipopolysaccharide (LPS) from *Escherichia coli* (400 µg; Sigma-Aldrich, St. Louis, MO, USA) was treated with ethyl-dimethylaminopropyl carbodiimide (270 µg) to convert carboxyl units to amine-reactive intermediates (final volume 500 µL), and then introduced to APTES-modified pSi microparticles to create stable amide bonds (2 h at 30 °C with agitation at 1,400 rpm), followed by washing. LPS-adsorbed (1 mg/mL) microparticles (3 × 10^8^), dried in a desicator, were loaded by capillary action as previously described using a solution made by mixing 50 µL OVA peptide (SIINFEKL (Genscript, Piscataway, NJ, USA); 10 µg/mL) with 50 µL (50 µg) amine-modified SPIONs (20 nm).

### Intracellular Interferon Assay

C57BL/6 (Charles River Laboratories, Inc.; Wilmington, MA, USA) BMDC were prepared by culturing bone marrow cells in granulocyte macrophage colony stimulating factor (20 ng/mL) and interleukin (IL)-4 (100 ng/mL) for 5 days. Splenic T-cells suspensions were prepared from OT-1 mice (The Jackson Laboratory, Bar Harbor, ME, USA) by passing cells through a 70 µm cell strainer and enriching T cells using a Dynal® Mouse T cell Negative Isolation kit (Invitrogen, Grand Island, NY, USA). BMDC were incubated with microparticles (20 to 1 ratio) for 2 h followed by 18 h co-culture with T cells at a one-to-one ratio (5 × 10^5^ each). Cells were permeabilized with Cytofix/Cytoperm™ solution and incubated with PE-conjugated anti-interferon gamma (IFN-γ) and FITC-conjugated anti-CD8 antibodies (BD Biosciences, Mountain View, CA, USA) for 30 min at 4 °C. Samples were analyzed using a LSRFortessa™ Flow Cytometer (BD Biosciences) using FACSDIVA™ software [20]. Statistical analysis was based on a two-tailed, equal variance t-test (n = 3 per group).

### Electron Microscopy Imaging of Particles and BMDC

SPIONs were suspended in water and dried on a formvar 400 mesh grid. Images were acquired using a JEOL 1200 transmission electron microscope (TEM) at 60 kV with digital images collected using a 1 k × 1 k Gatan BioScan camera Model 792.

BMDC were cultured on poly-l-lysine glass slides with LPS-bound pSi microparticles (1:20 ratio) in the presence of 10 µg/mL SIINFEKL peptide for 3 h at 37 °C. OT-1 splenic T cells were introduced at a ratio of 2:1 (BMDC:T) and cells were incubated for an additional hour. Following fixation in 2% glutaraldehyde in 0.1 M cacodylate buffer, pH 7.4, cells were incubated in a mixture of osmium tetroxide (OsO_4_) and 1% potassium ferrocyanate in 0.1 M cacodylate buffer for 30 min at 48 °C. Cells were then dehydrated and embedded with a mixture of epon and araldite. Ultrathin sections were counter-stained with uranyl acetate and imaged using a JEOL 1210 transmission electron microscope equipped with an AMT Imaging System. Gamma adjustments were made to the micrographs to enhance image contrast and brightness.

For surface topography imaging by scanning electron microscopy (SEM), BMDC were plated in 24 well plates containing 5 × 7 mm silicon chip specimen supports (Ted Pella, Inc., Redding, CA, USA) at 1 × 10^5^ cells per well. The next day, cells were incubated with pSi microparticles for 3 h at 37 °C (1:10; cell:microparticle ratio). BMDC were either fixed and dehydrated as previously described [18, 21] for SEM imaging or further incubated with OT-1 T cells for an additional h then processed for imaging. Cells were sputter-coated with a 4 nm layer of platinum/palladium (80:20) using a Cressington Sputter Coater 208 HR (Ted Pella, Inc., Redding, CA, USA). SEM images were acquired under high vacuum, at 10 kV, spot size 3.0, using a Nova™ NanoSEM (FEI Company, Hillsboro, OR, USA). Images were pseudo-colored and gamma levels were adjusted to enhance image contrast and brightness.

### Statistical Analysis

Relaxivity data is expressed as the slope of the relaxation rate *vs*. concentration. The slope was calculated by best fit linear regression analysis using the least squares method. 95% confidence intervals were generated using the standard error of the slope (SE_slope_), given by the following equation:

$${\text{SE}}_{\text{slope}}=\text{sqrt}[\Sigma {\left(\text{yi}-\hat{y}i\right)}^{2}/(n-2)]/\text{sqrt}[\Sigma {\left(\text{xi}-x\right)}^{2}]$$

where yi is the value of relaxation rate for instance i, ŷi is estimated value of the relaxation rate for observation i, xi is the observed value of the concentration for observation i,×is the mean of the concentration, and n is the number of data points (n = 5). The 95% confidence interval was then calculated using the formula

$$\text{CI}=\pm {\text{SE}}_{\text{slope}}\times 1.96$$

Regression analysis was done using a single set of relaxation rate measurements *vs*. concentration for each of the free or encapsulated SPION particle preparations analyzed. Significance was determined with non-overlapping confidence intervals.

## 3. Results

### 3.1. Hybrid Particles

**Figure 1:**
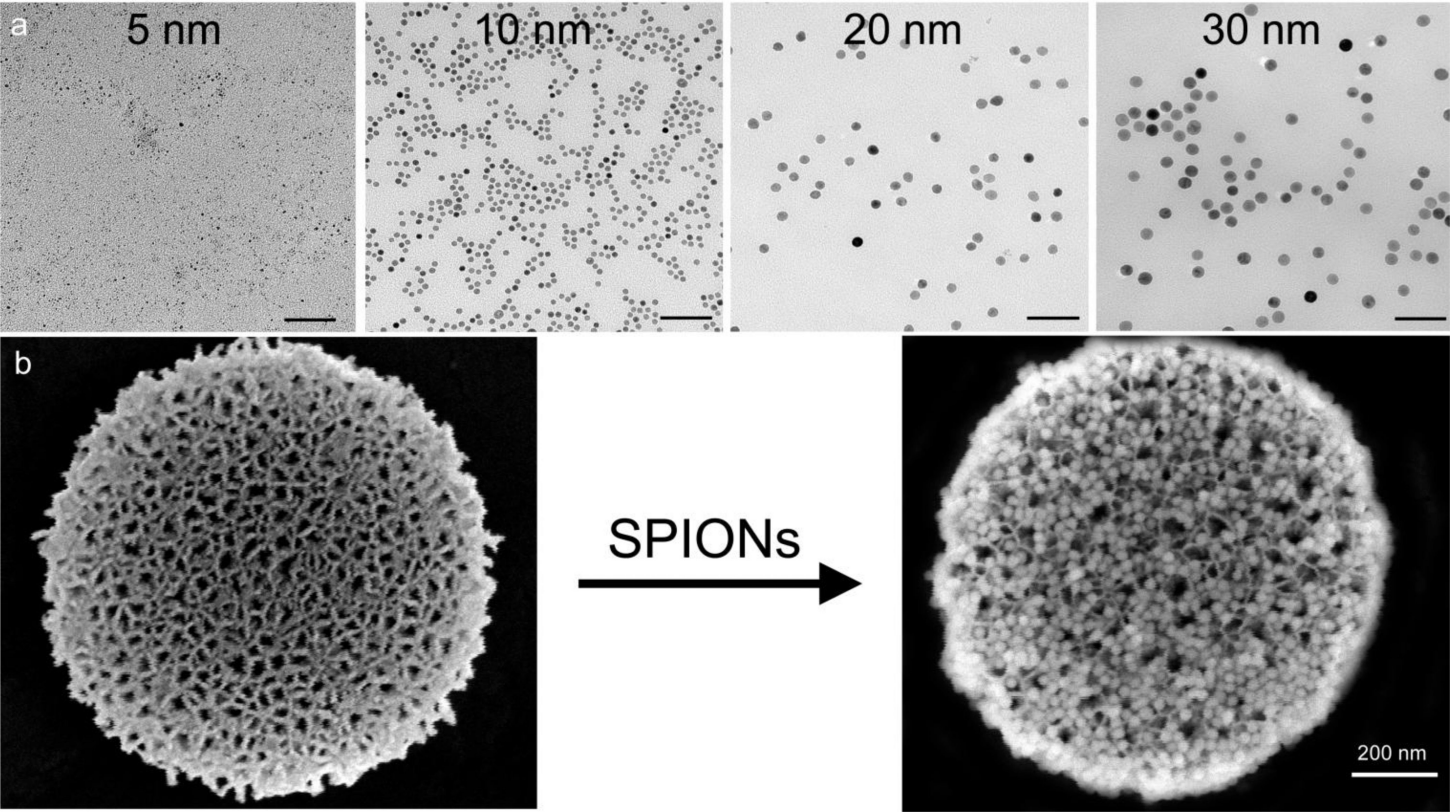
Electron micrographs of single and hybrid particles. (**a**) TEM images of superparamagnetic iron oxide nanoparticles (SPIONs) with core sizes of 5, 10, 20, and 30 nm on 400 mesh formvar carbon grids, imaged at 200,000× direct magnification (bars = 100 nm). (**b**) SEM images of discoidal porous silicon (pSi) microparticles, unloaded (left) or loaded with 30 nm SPIONs (right with image periphery pseudo-colored black).

TEM images of SPIONs, with core sizes ranging from 5–30 nm, are presented in Figure 1a. The particles are uniform in size and distribution. SEM images confirm that pSi microparticles are discoidal in shape with dimensions of 1,000 × 400 nm (Figure 1b). The porosity of the microparticles is 70%–80%, with each microparticle having a volume of 0.2 µm^3^, with an average surface pore diameter of 50 nm. Abundant loading of SPIONs by capillary action into the pSi matrix is shown in Figure 1b.

### 3.2. SPION Magnetic Properties

**Figure 2:**
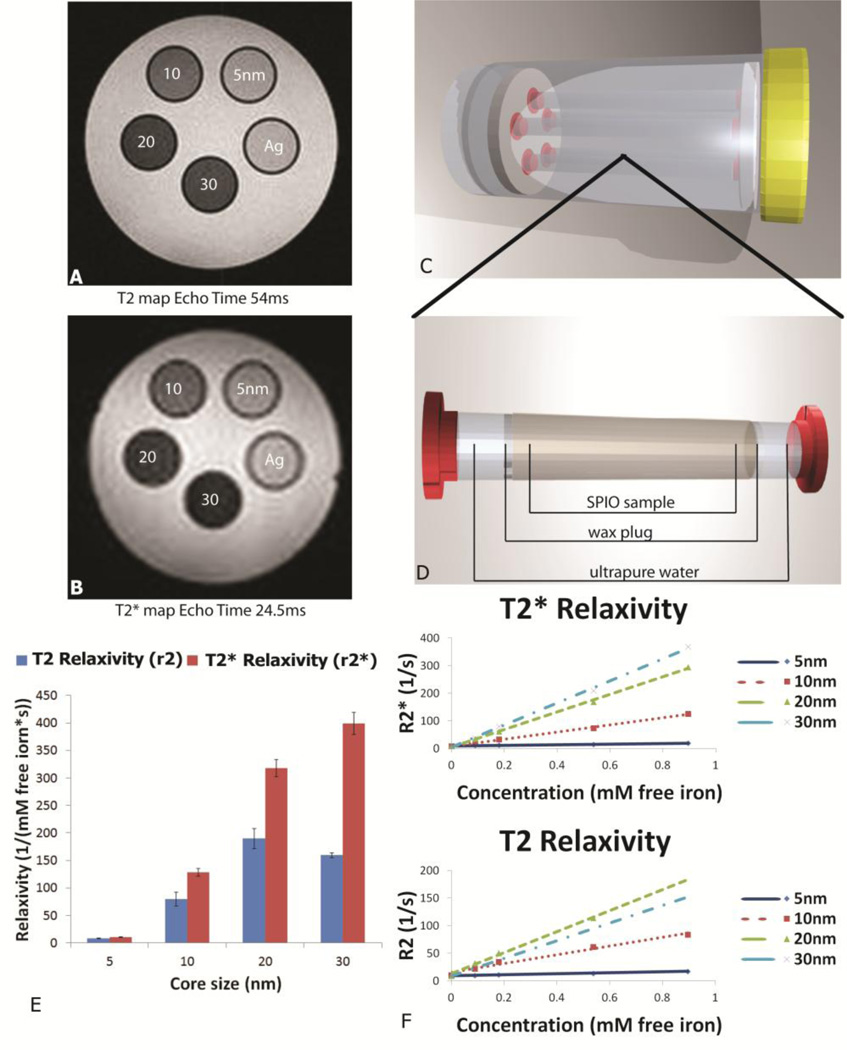
Impact of SPION core size on negative (T2) contrast and proton relaxivity. (**A**) T2 MR image, echo time 54 ms, of an axial slice through agarose phantoms containing 5, 10, 20, and 30 nm core size SPIONs with a pure 1% agarose control (labeled Ag). All SPION phantoms contained an equivalent amount of iron oxide at a concentration of 0.05 mg/mL. (**B**) T2* MR image, echo time 24.5 ms, of an axial slice through the same phantoms as described in (**A**). (**C**) 3D model of apparatus used to image particle phantoms in MRI. Phantoms were suspended in ultrapure water and held away from the plastic ends of the sample holder to minimize artifacts created by abrupt changes in magnetic susceptibility between the phantoms and surrounding medium. The sample tubes and holder were constructed of polystyrene and polypropylene, respectively. (**D**) NMR sample tube with SPIONs suspended between 2 wax plugs and surrounded by DI water. (**E**) Comparison of r2 and r2* values for 5, 10, 20, and 30 nm core size SPIONs with 95% CI displayed as error bars. (**F**) Plot of the relaxation rate *vs*. concentration of iron expressed in units of mM free iron. The slope of the relaxation rate *vs*. concentration curve represents the relaxivity, r2 and r2*, expressed as 1/(mM × s).

Differences in negative contrast in T2 and T2*-weighted images of SPIONs were visible in phantom preparations containing SPIONs of increasing core size (Figure 2a,b), despite the fact that the actual content of free iron remained constant. Devices used to create phantoms are shown pictorially in Figure 2c,d. SPIONs, suspended in 1% agarose within NMR tubes, were placed in a 5 specimen holder which was then filled with relaxed water. As predicted by MAR theory, SPIONs showed a trend of increasing relaxivity with increasing core size using both T2 and T2* imaging sequences (Table 1 and Figure 2e,f). This trend was demonstrated up to a core size of 20 nm for T2-weighted measurements. In contrast, a plateau was not reached for particles at or below 30 nm for T2*-weighted measurements (Figure 2e).

### 3.3. Impact of pSi Encapsulation on SPION Magnetic Properties and Cellular Uptake

**Figure 3:**
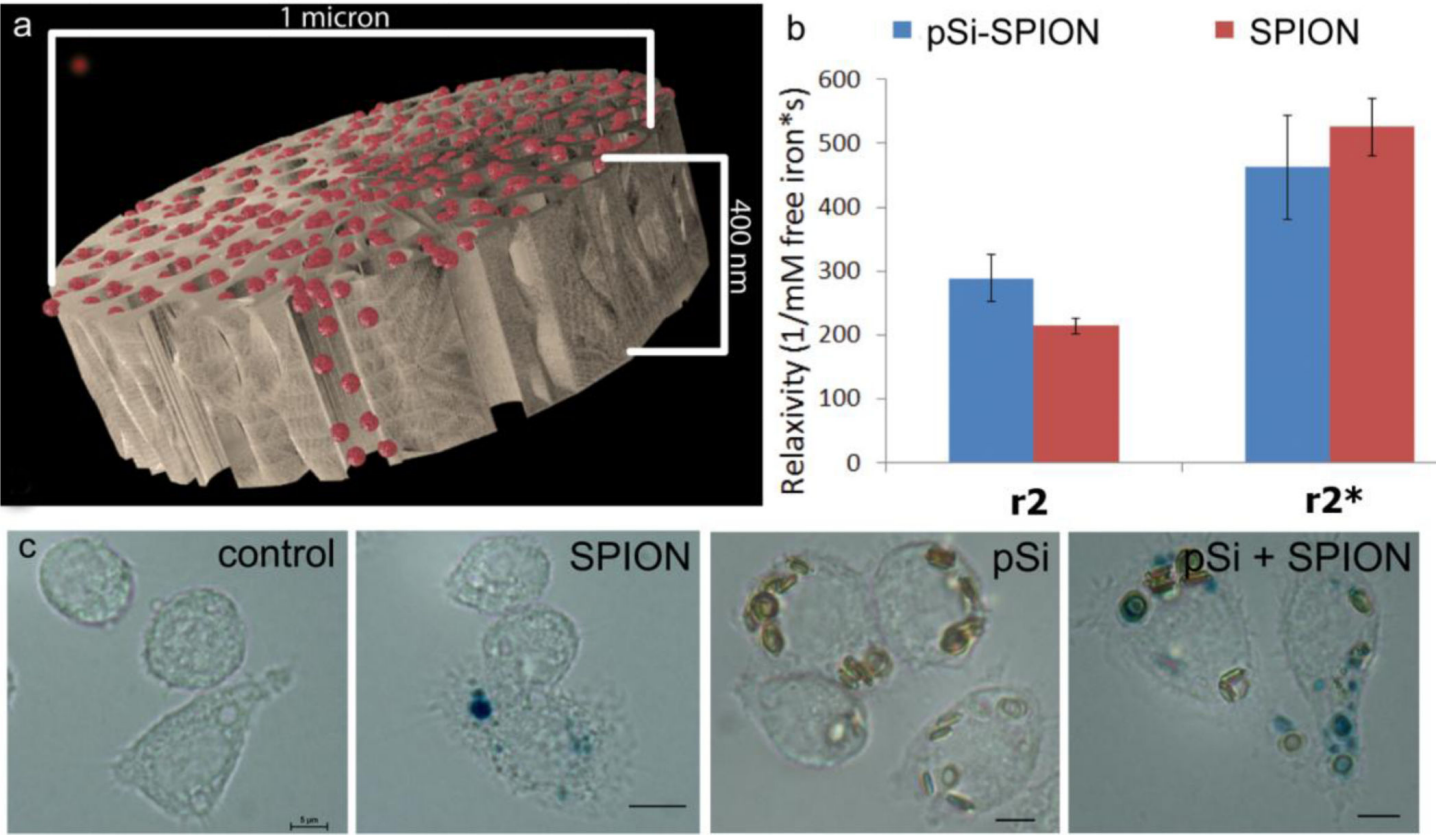
Influence of SPION encapsulation in the pores of pSi microparticles on MRI contrast enhancement and cellular uptake. (**a**) Computer simulated image showing SPIONs (red) loaded into a discoidal pSi microparticle. (**b**) r2 and r2* relaxivity of free or encapsulated 30 nm SPIONs in agarose phantoms. Relaxivity was calculated using the slope of the relaxation rate *vs*. concentration. Error bars represent 95% confidence intervals. (**c**) Bright field micrographs of J774 mouse macrophages following 24 h incubation with free (SPION) or pSi encapsulated SPIONs (pSi + SPION), or unloaded pSi microparticles (pSi). Cells were stained with Prussian blue to visualize localization of iron oxide.

Based on SDR thoery, it was hypothesized that SPION encapsulation in a pSi microparticle would increase T2* relaxivity based on particle clustering within the silicion matrix. Loading of 30 nm SPIONs into discoidal pSi microparticles, confirmed by SEM imaging (Figure 1b) is illustrated in Figure 3a. T2 relaxivity (r2) values for encapsulated and free SPIONs embedded in agarose phantoms were 288 mM^−1^s^−1^ (95% CI 251–325) and 214 mM^−1^s^−1^ (95% CI 202–225), respectively (Figure 3b). While this represents a statistically significant increase in T2 relaxivity with encapsulation, the magnitude of the increase is not predicted to be great enough to be clinically significant. T2* relaxivity values for encapsulated and free SPIONs were statistically similar, at 462 mM^−1^s^−1^ (95% CI 381–543) and 525 mM^−1^s^−1^ (95% CI 480–570), respectively.

Macrophages are capable of internalizing both nano and micro particles [19, 22, 23]. In Figure 3c, we demonstrate uptake of free and pSi encapsulated SPIONs by J774 mouse macrophages following 24 h incubation at 37°C. Previously we reported that silicon microparticles remain entrapped in endolysosomes, while the fate of the secondary SPIONs is influenced by the surface properties of the nanoparticles. In Figure 3c, SPIONs, stained with Prussian blue, are shown both co-localized and distinct from the pSi microparticles.

### 3.4. Accumulation of SPIONs in the Spleen

**Figure 4:**
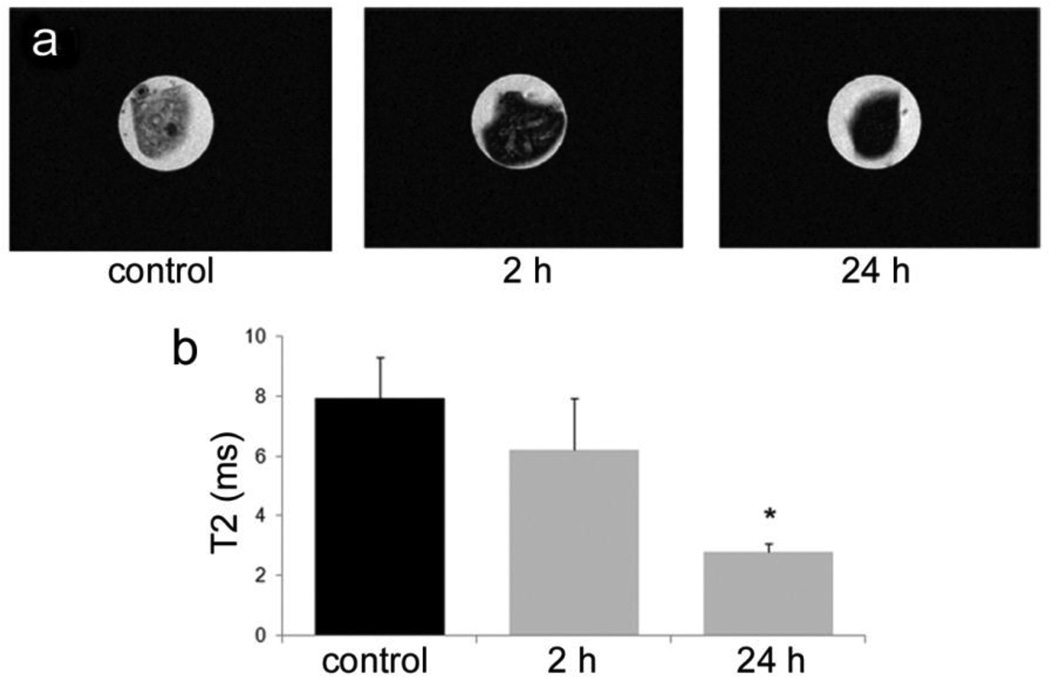
Time-dependent accumulation of intravenously injected SPIONs in the spleen of mice. (**a**) Spleen, recovered from mice at 2 and 24 h post intravenous injection with 20 nm SPIONs, were suspended in agarose phantoms and imaged using a 7T MR scanner. T2-weighted MR images are presented, along with the spleen from a control animal. (**b**) T2 values of the imaged spleen are displayed graphically (*p < 0.007 compared to control).

Nanoparticles are being investigated as vaccine delivery vehicles for transporting immune modulators and antigens to APCs. In this study, we examined trafficking of SPIONs to lymphoid tissue based on enhanced negative contrast in the spleen. Mice, given intravenous injections of SPIONs (100 µg Fe), were sacrificed at 2 and 24 h post injection and axial T2-weighted images were acquired for spleen suspended in phantoms (Figure 4a). Nanoparticle accumulation was time-dependent, with signal intensity in the spleen being significantly lower for the animal sacrificed at 24 h compared to the control animal (*p* < 0.007) (Figure 4b).

### 3.5. Trafficking of Nanoparticles to Lymphoid Tissues

**Figure 5:**
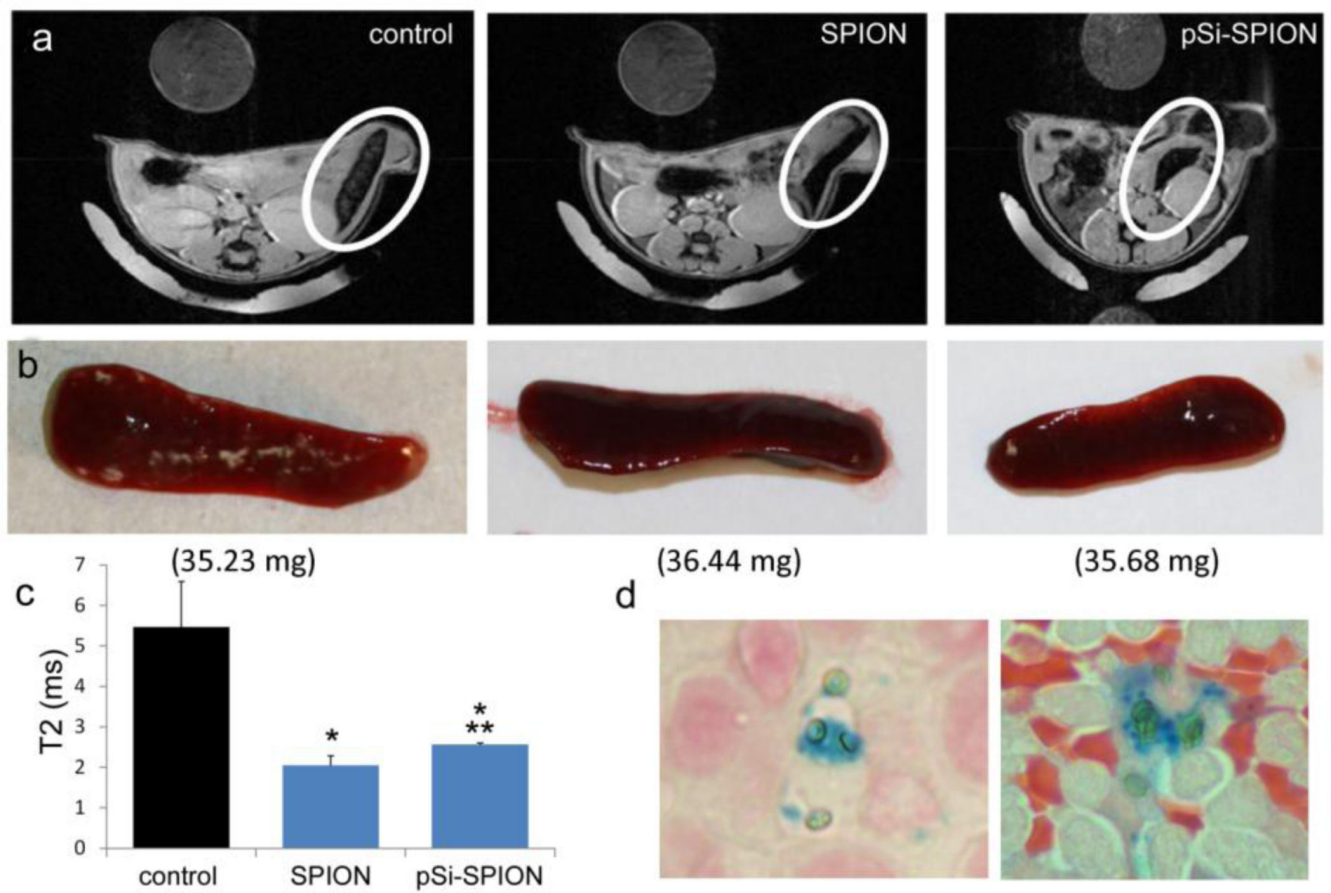
Influence of SPION encapsulation in pSi microparticles on T2 contrast in the spleen. (**a**) T2-weighted axial images of female nu/nu mice injected with either PBS (left image), 30 nm SPIONs (100 µg Fe) (middle), or pSi-encapsulated 30 nm SPIONs (100 µg Fe; right image) 24 h post injection. Spleens are indicated with white circles, with the corresponding gross tissues and weights shown below for each respective group (**b**). An internal SPION reference standard (0.01 mg/mL) (dark circle located at the top of each MRI image) was included in each image. (**c**) Measured *in vivo* T_2_ values for control, free (SPION) and encapsulated (pSi SPION) SPIONs. Data represents the mean of 5 ROIs in unique slices and the standard deviation (*p* < 0.02; * compared to control; ** SPION *vs*. pSi SPION). (**d**) Histological specimens of dissected spleens isolated from mice treated with pSi-encapsulated SPIONs (24 h post injection) labeled with Prussian blue and Nuclear Fast Red.

To study the impact of particle presentation on trafficking of SPIONs to lymphoid tissue, axial T2-weighted images of nude mice following intravenous injections of free or pSi-encapsulated SPIONs (100 µg Fe) were acquired 24 h post injection (Figure 5a). Enhanced negative contrast due to SPION accumulation in the spleen was observed visually in mice injected with either free SPIONs or pSi-encapsulated SPIONs. Excised spleen was darker from SPION-treated compared to the control mice (Figure 5b). Average T2 contrast was significantly enhanced in the spleens of SPION-treated mice compared to control animals, with T2 values supporting greater accumulation of free SPIONs compared to hybrid particle SPIONs (Figure 5c). Co-localization of SPIONs and pSi microparticles in the spleen was confirmed by Prussian blue staining of histological specimens (Figure 5d).

### 3.6. Hybrid Particles as Vaccines Platforms

**Figure 6:**
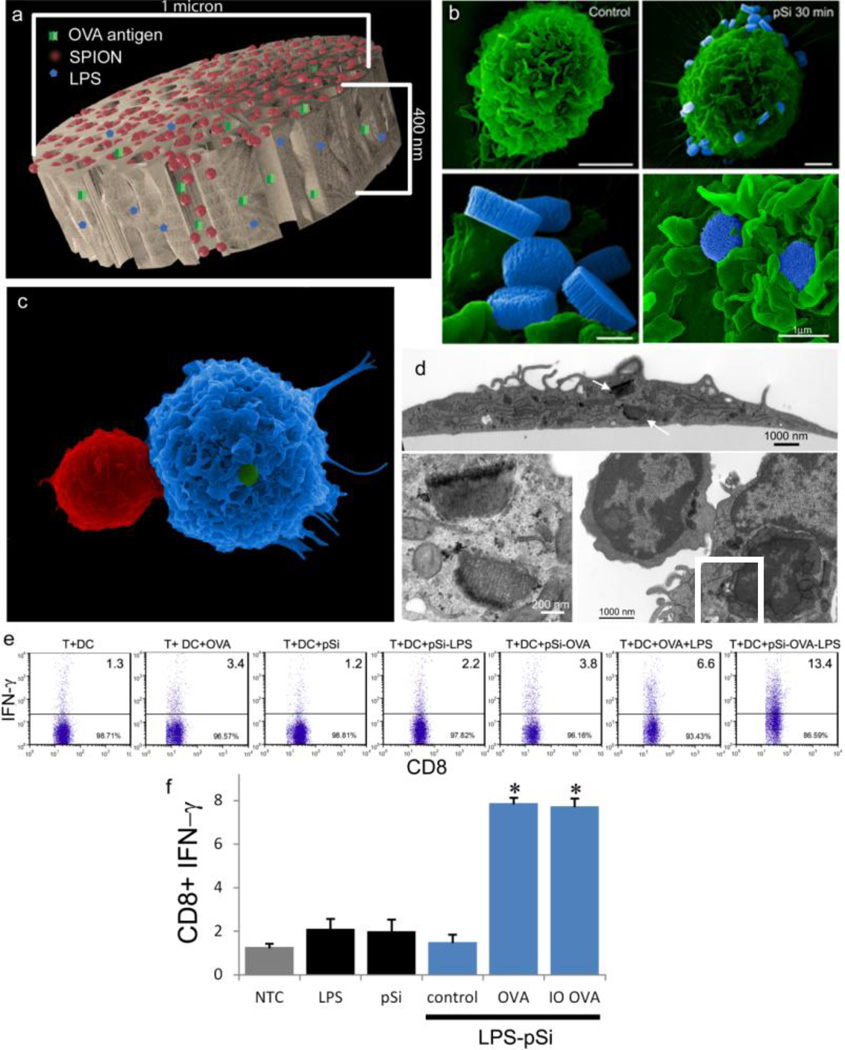
Hybrid particles as adjuvants for antigen presentation. (**A**) Computer simulated image showing SPIONs (red), ovalbumin peptide (OVA; green) and lipopolysaccharide (LPS) (blue) loaded into a pSi microparticle. (**B**) SEM images showing control and pSi microparticle-treated bone marrow-derived dendritic cells (BMDC) (30 min; bars 3 (left) and 2 µm (right); 10–13 k mag). High magnification images show a group of microparticles on the cell surface (bottom left, bar 500 nm; 50 k mag) and two microparticles engulfed in BMDC membrane folds during early uptake (bottom right; bar 1 µm; 40 k mag). (**C**) SEM image showing particle adjuvant-induced association of a T cell with the stimulated BMDC. (**D**) TEM image showing a BMDC following treatment with LPS-pSi microparticles (3 h; bar 2 µm). The boxed region is shown at higher magnification in the lower left image, showing microparticles surrounded by cytoplasmic constituents. The bottom right image shows a T cell bound to a BMDC following treatment with adjuvant particles. (**E**) Flow cytometry dot blots showing the percent of interferon-γ (IFN-γ) positive OT-1 CD8^+^ T cells following 4 h treatment with particle-stimulated (24 h) C57BL/6 BMDC (reproduced courtesy of Meraz *et al*. [24]). (**F**) C57BL/6 BMDC were incubated with various formulations of the hybrid particle vaccine then introduced to OT-1 T cells for 4 h, followed by analysis for intracellular IFN-γ production by flow cytometry (n = 3). Controls included free LPS, unloaded pSi microparticles, and LPS-pSi microparticles, either empty (control) or loaded with OVA peptide, in the absence or presence of 20 nm SPIONS (abbreviated OVA and IO OVA) (*p < 0.0007).

Using particles as carriers for adjuvants and antigens simultaneously stimulates APCs and leads to internalization of antigen, with sustained release of the antigen potentially expanding the duration of antigen presentation. We previously demonstrated that multivalent presentation of toll-like receptor (TLR) ligands on the surface of carrier silicon microparticles activates DC to a greater extent than free ligand, leading to enhanced DC uptake, and increased migration of DC to the draining lymph node [24]. In this study, we have added a model antigen (OVA) and secondary nanoparticles (*i.e.*, SPIONs) to the complex to direct the immune response and to enable tracking of the vaccine (Figure 6a). As stated, BMDC internalize TLR ligand bound pSi microparticles. In Figure 6b, a control BMDC is shown pseudo-colored in green. To the right, a second BMDC is shown with blue-pseudo-colored pSi microparticles bound to the cell membrane. The boxed region in the image is shown at higher magnification in the bottom row of images to highlight microparticle orientation and early stages of phagocytosis. Membrane veils can be seen wrapping around the microparticles in the bottom right image of Figure 6b.

To study antigen presentation by microparticle (LPS-OVA-pSi)-treated C57BL/6 BMDC to OT-1 T cells, we first used SEM to demonstrate particle-induced cellular interactions (Figure 6c). The large DC is seen bound to the smaller T cell. Confirmation of microparticle internalization within BMDC was achieved using TEM imaging. The arrows in Figure 6d indicate microparticles within the cell, with the internalized particles (boxed region) shown in the higher magnification image in the lower left image. A close-up view of T cell-DC binding is shown in the lower right image. Following particle activation of BMDC and incubation with OT-1 T cells, intracellular IFN-γ production was evaluated in CD8^+^ T cells by flow cytometry. Figure 6e, adapted from Meraz *et al*. [24], shows that LPS enhances OVA induced IFN-γ production by T cells, and that presentation of LPS by particles further increases T cell activation. In a novel experiment (Figure 6f), incubation of BMDC with LPS-pSi microparticles loaded with OVA peptide, in the presence and absence of SPIONs, stimulated IFN-γ production by CD8^+^ T cells.

## 4. Discussion

Previously, we demonstrated that loading of SPIONs into pSi microparticles creates hybrid particles with magnetic properties, with increasing payloads of SPIONs inducing greater negative contrast in MR images [19]. During the loading process, increases in the concentration of SPIONs introduced to dry pSi microparticles increases nanoparticle loading in the silicon pores, with a plateau reached at concentrations above 1 mg/mL [19] Theoretically, the pore volume of each 1,000 × 400 nm pSi microparticle is about 0.2 µm^3^permitting loading of up to 3.8 × 10^5^ 10 nm nanoparticles. Empirically, we previously demonstrated loading of 0.3 pg of 10 nm SPIONs per pSi microparticle, corresponding to approximately 1 × 10^5^ SPIONs [25].

This manuscript expands on previous work by showing the impact of SPION size and encapsulation in the pores of a silicon microparticle on negative contrast enhancement. As stated previously, relaxation effects of SPIONs are governed by their magnetic properties which are determined by particle size, shape and morphology. As reported by others, we demonstrated that increasing core sizes generates superior relaxivities [26]. At the level of external magnetic field strength of most clinical scanners (1.5–3 Tesla), the induced magnetization of SPION contrast agents is very close to the asymptotic upper limit, referred to as the induced magnetization at saturation. As the diameter of SPIONs is increased, the induced magnetization at saturation also increases [27], causing increases in T2 and T2* relaxivity [28]. Plotting relaxation rates (T2, T2*) against concentration (mM free iron) generates slopes which yield the concentration-independent relaxivities (r2 and r2*). In this study, using a 7 Tesla scanner, we demonstrate that SPION relaxivities increase with particle diameters ranging from 5 to 30 nm, with iron saturation effects becoming apparent for r2 values for particles 20 nm and higher, and r2* showing a further increase as the core size increases from 20 to 30 nm.

Accumulation of ultrasmall SPIONs in the phagosomes of macrophages has been shown to enhance decreases in the signal intensity of T2*-weighted images due to shortening of T2 and T2*, with tissue clustering leading to additional T2* shortening effects [29]. Similarly, spatial confinement of SPIONs in larger nanoparticles, such as micelles, has been shown to produce strong field gradients leading to increased transverse relaxivities [30, 31]. Kinsella *et al*. [32] reported that encapsulation of 9 nm Fe_3_O_4_ in 16 nm silicon pores led to an increase in magnetic strength causing an increase in transverse relaxivity. In this study, encapsulation of 30 nm SPIONs within a pSi matrix (50 nm pores) caused a slight decrease (*p* < 0.05) in T2 transverse relaxation, but no significant impact on T2* values. There was no increase in the ratio of R2* to R2 with compartmentalization of SPIONs in pSi, as predicted by SDR theory. In contrast to previous work by our group [33] that showed large alterations in T1 contrast with encapsulation of gadolinium within pSi microparticles, the small enhancement in T2 relaxivity due to encapsulation of SPIONs in pSi microparticles is not likely to add significant clinical benefit on its own. However, benefits of using hybrid particle complexes, as opposed to free SPIONs, are due to incorporation of additional biologically active agents, such as immune modulators and antigens, achieving delivery of both imaging and therapeutic agents to the same target cells.

As previously demonstrated by others, free SPIONs accumulate predominately in the liver and spleen following intravenous administration [34], with size and surface modification of the nanoparticles impacting biodistribution [35]. Time-dependent accumulation of SPIONs in the spleen with time (Figure 4) was confirmed, with greater levels of SPIONs detected at 24 h compared to 2 h by MRI. Based on higher accumulation at the later time point, 24 h was used for *in vivo* MR imaging studies designed to compare accumulation (*i.e.*, detection) of free verses pSi encapsulated SPIONs. Detection of free SPION accumulation in the spleen was slightly superior to pSi-encapsulated SPIONs following intravascular injection. Future work will examine particle accumulation in the draining lymph nodes following subcutaneous injection to explore the impact of mode of transportation (*i.e.*, cell-based transport for larger particles verses exit from capillaries and trafficking through the lymphatic system for smaller particles [17]) on particle accumulation in lymphatic tissue. In addition, we previously demonstrated that particle surface charge alters accumulation of microparticles in the spleen following intravenous administration, with cationic microparticles accumulating preferentially over anionic microparticles [21]. Since both particle charge and macromolecule binding (e.g., opsonization or ligand conjugation) to the particle surface influence biodistribution, future studies will also examine the impact of loading microparticles with antigens and immune modulators on trafficking of vaccine components to lymphatic tissue.

As demonstrated previously, we show abundant adhesion and uptake of pSi microparticles by BMDC. Loading of antigen and immune modulators (*i.e.*, LPS) stimulates antigen driven interactions between OT-1 T cells and particle-treated BMDC, with enhanced production of IFN-γ in activated CD8^+^ T cells. Incorporation of SPIONs into the particle complex did not alter stimulation of T cell immunity, supporting the use of the hybrid particles for the development of immunotherapeutics.

## 5. Conclusions

This study supports well-established theories of size-dependent enhanced transverse relaxivity of surrounding water protons by SPIONs. While particle presentation did not have a major impact on contrast enhancement, a hybrid particle system did permit presentation of a diverse payload for a theranostic approach to vaccination. Our hybrid particle vaccine presents contrast agents and antigens in combination with pattern-associated molecular pattern molecules for tracking and stimulation of targeted immune responses. Preliminary data supports antigen presentation by particle-treated DC and stimulation of cellular immune responses. Future work will test the efficacy of the hybrid particle complex for generation of *in vivo* immune responses.

## Figures and Tables

**Table 1:** Relaxivity values r2 and r2* for each core size expressed as mM^−1^s^−1^ (n = 5 data points except as indicated).

Core size (nm)	T2 Relaxivity (r2)		T2* Relaxivity (r2*)	
5	8.824	+/− 0.50	10.556	+/− 0.90
10	79.56	+/− 12.30	128.69	+/− 6.97
20 *	189.89	+/− 18.64	317.79	+/− 15.58
30 **	159.62	+/− 4.09	399.37	+/− 20.28

* r2 values calculated using n = 4 data points;

** r2 values calculated using n = 3 data points.
